# Changing epidemiology of COVID-19 

**DOI:** 10.3205/dgkh000362

**Published:** 2020-11-06

**Authors:** Benedikt M.J. Lampl, Bernd Salzberger

**Affiliations:** 1 Public Health Department Regensburg, Germany; 2 University of Regensburg, Germany; 3 Department of Infection Control and Infectious Disease, University of Regensburg, Germany

**Keywords:** COVID-19, epidemiology, PCR, asymptomatic cases, testing strategies

## Abstract

**Background:** We analyzed the epidemiology of COVID-19 in Regensburg after the first wave ended in June 2020 and compared it with patients’ characteristics and symptoms in late summer/early autumn 2020.

**Methods:** Retrospective analysis of epidemiological data from Regensburg (city/county) on age and initial symptoms as reported during case investigation for containment. Observed periods: March 7, 2020 to June 6, 2020 and August 12, 2020 to October 9, 2020.

**Results:** The proportion of asymptomatic persons who tested positive for SARS-COV-2 in the second period was 55% (286 of 520 cases), whereas during the first wave from March to June 2020 this percentage was 14.4% (169 of 1,170 cases). A comparison of typical symptoms shows that the most common symptoms of COVID-19 in the first wave (cough, fever and generally feeling ill) were less often reported in the second period: cough 14% vs. 42%, fever 17% vs. 38%, general signs of illness 14% vs. 22% in the second vs. first period, respectively overall cases were younger in the second period, the median age of asymptomatic cases was comparable in both periods. The case fatality rate for the first period was 2.1%, in the second it was 0.2%.

**Discussion:** The epidemiological situation in the second period is different from that during the first wave. We observed a considerable proportion of questionable cases in August/September 2020 (asymptomatic cases, high ct values, often only detection of one gene). False positive cases/non-contagious cases have to be taken into account for this period. On-demand or free-of-charge testing for asymptomatic persons will lower the positive predictive value of tests and place a high burden on finite capacities.

## Zusammenfassung

**Hintergrund:** Es wurden Patientencharakteristika und Symptome von COVID-19 in Regensburg in der ersten Welle und nach dem Ende der ersten Welle verglichen.

**Methoden:** Retrospektive Analyse epidemiologischer Daten aus Regensburg (Stadt/Landkreis) zu Alter und Symptomen bei Erstmeldung des Falls; beobachtete Zeiträume 07.03.2020 bis 06.06.2020 und 12.08.2020 bis 09.10.2020.

**Ergebnisse:** Der Anteil asymptomatischer Personen, von denen nicht mindestens ein Symptom einer akuten Infektion angegeben wurde, die jedoch positiv auf SARS-COV-2 getestet wurden, betrug im zweiten Zeitraum 55% (286 von 520 Fällen), während dieser Prozentsatz während der ersten Welle von März bis Juni 2020 bei 14,4% lag (169 von 1.170 Fällen). Ein Vergleich typischer Symptome zeigt, dass die häufigsten Symptome von COVID-19 in der ersten Phase (Husten, Fieber und allgemeines Krankheitsgefühl) in der zweiten Periode auffällig seltener berichtet wurden: Husten 14% vs. 42%, Fieber 17% vs. 38 %, allgemeine Anzeichen einer Krankheit 14% gegenüber 22%. Insgesamt waren die Betroffenen in der zweiten Periode jünger, das Durchschnittsalter der asymptomatischen Fälle war in beiden Perioden vergleichbar. Die Sterblichkeitsrate (case fatality rate, CFR) für den ersten Zeitraum betrug 2,1%, für den zweiten 0,2%.

**Diskussion:** Die epidemiologische Situation im zweiten Beobachtungszeitraum ist nicht mit der ersten Welle vergleichbar. Im August/September 2020 konnte ein erheblicher Anteil fraglicher Konstellationen beobachtet werden (asymptomatische Fälle, hohe ct-Werte, oft nur Nachweis eines Gens). Falsch positive Fälle/nicht ansteckende Fälle müssen für diesen Zeitraum angenommen werden. Anlasslose Tests bei asymptomatischen Personen senken den positiven Vorhersagewert und belasten die vorhandenen Kapazitäten.

## Background

COVID-19 has rapidly spread all over the world [1]. During a first high incidence period (“first wave”) from March to May 2020 it affected many countries severely [2]. As symptoms are non-specific and typical for respiratory infections and the clinical spectrum of COVID-19 ranges from asymptomatic to severe or even fatal, disease containment is necessary, albeit difficult [3]. Thus, strategies to control its spread had to be established.

In Bavaria, a liberal strategy of PCR testing was implemented at the end of the summer holidays; tests were offered free of charge even to asymptomatic citizens, and testing of travelers from risk areas was obligatory [4], [5]. 

During the first wave, a high number of undiagnosed cases (under-reporting) probably existed, in addition to a comparably high number of confirmed severe and fatal cases. The number of these undetected cases was calculated with different mathematical models for different countries [6]. In contrast, in the second (current) wave, a lower proportion of severe cases has been observed so far. Furthermore, it was conspicuous that the Public Health Department had to deal with very heterogenous laboratory results: a considerable number of samples showed only one positive gene (usually the two-step test detects two genes: e-gene, n-gene or RdRP-gene [7]) and threshold cycle (ct) values above 35, representing a low viral load, if any.

In order to correlate these findings with clinical severity, we analyzed cases of the first wave from March 7, 2020 to June 6, 2020 (first wave) and cases from August 12, 2020 (when numbers of cases began to increase after a low prevalence in June/July) to October 9, 2020 in Regensburg (city/county) in terms of asymptomatic cases and distribution of symptoms in symptomatic cases. 

## Methods

Epidemiological data from Regensburg (city/county) on initial symptoms as reported during case investigation for laboratory confirmed (SARS-CoV-2 PCR: positive) cases were compared for the periods of March 7, 2020 to June 6, 2020 vs. August 12, 2020 to October 9, 2020. Symptoms were analyzed according to categories for reporting to the National Center of Infectious Disease Control (Robert Koch-Institut, RKI). Case definitions were applied according to the RKI [8]. An epidemic curve depicts all reported cases from March 7, 2020 (first reported case in Regensburg). Our database (Äsculab21, accessed October 10, 2020) was searched for cases and one of the following symptoms/conditions at the time of case investigation: cough, fever, general signs of illness, sore throat, rhinitis, dyspnoea, diarrhoea, taste disorder, hypogeusia, pneumonia, tachycardia, tachypnoea, ARDS, pneumonia requiring ventilation. 

Statistical analysis: *Microsoft Excel 2016* and a web-based calculator for the Wilcoxon rank-sum test were used [9]. 

## Results

**Figure 1:**
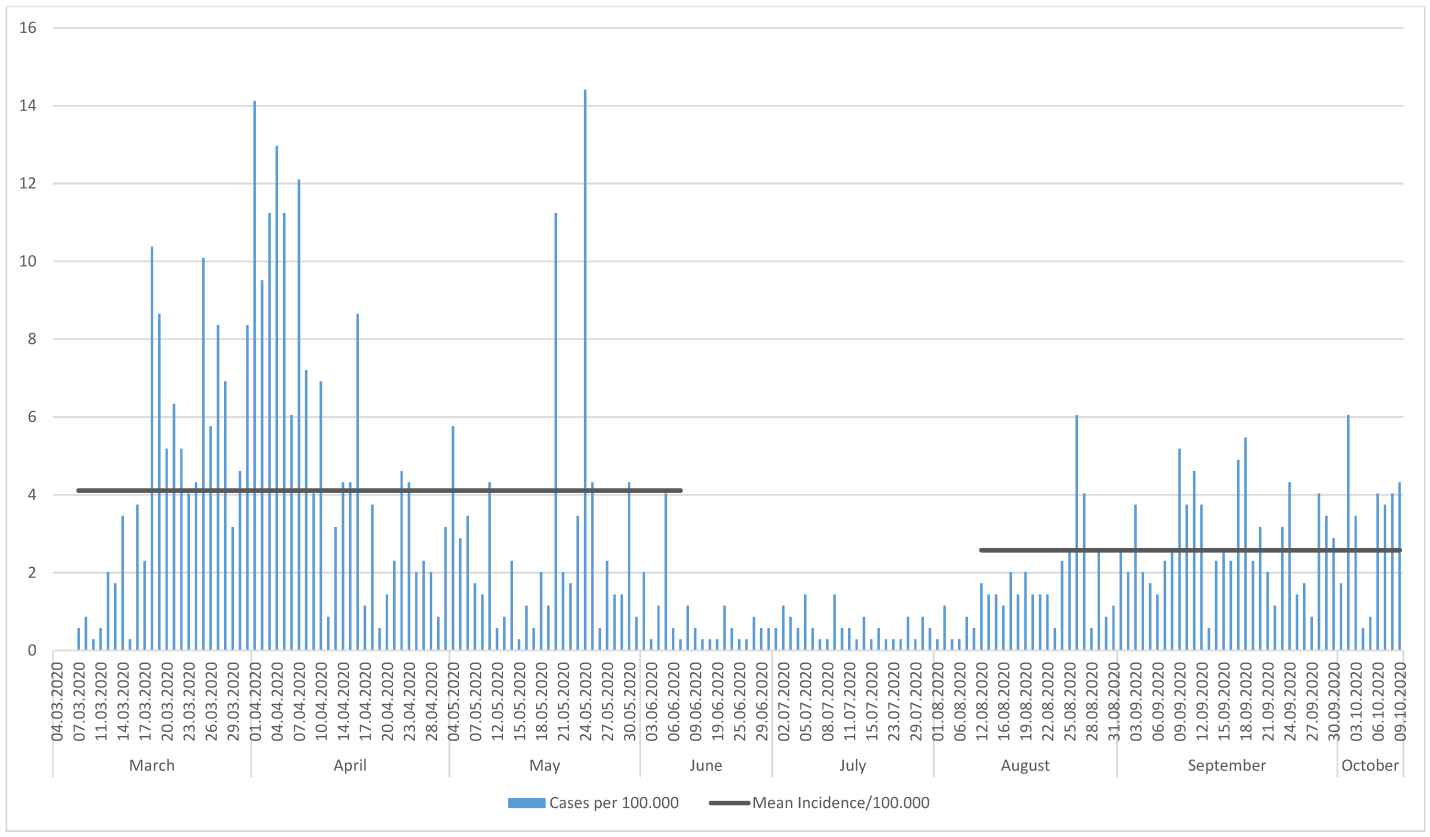
Epidemic curve, mean incidences calculated for the periods: March 7, 2020 to June 6, 2020 and August 12, 2020 to October 9, 2020

In the first period, a total of 1,170 cases were reported (epidemic curve, Figure 1 (Fig. 1)) with a mean incidence of 4.1/100,000/day. As of mid-August, an increase in case numbers was obvious after a phase of low numbers in June and July; this increase was associated with the end of the summer holidays and the implementation of the Bavarian testing strategy (wide availability of testing and obligatory testing for travelers from risk areas). From 12/08/2020 until 09/10/2020, 520 cases were reported, with a mean incidence of 2.6/100,000. 

**Figure 2:**
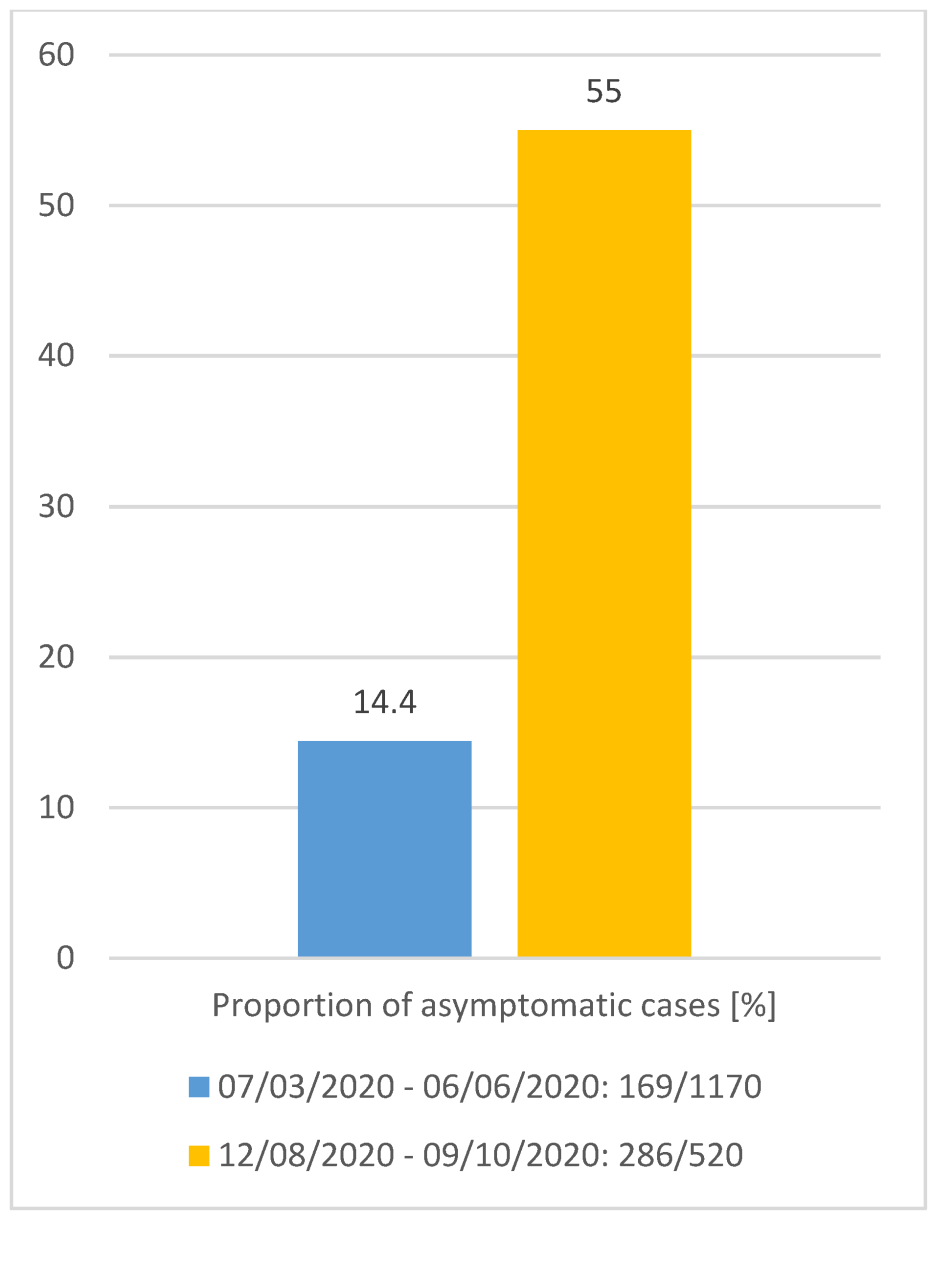
Percentage of initially asymptomatic cases in comparison

**Figure 3:**
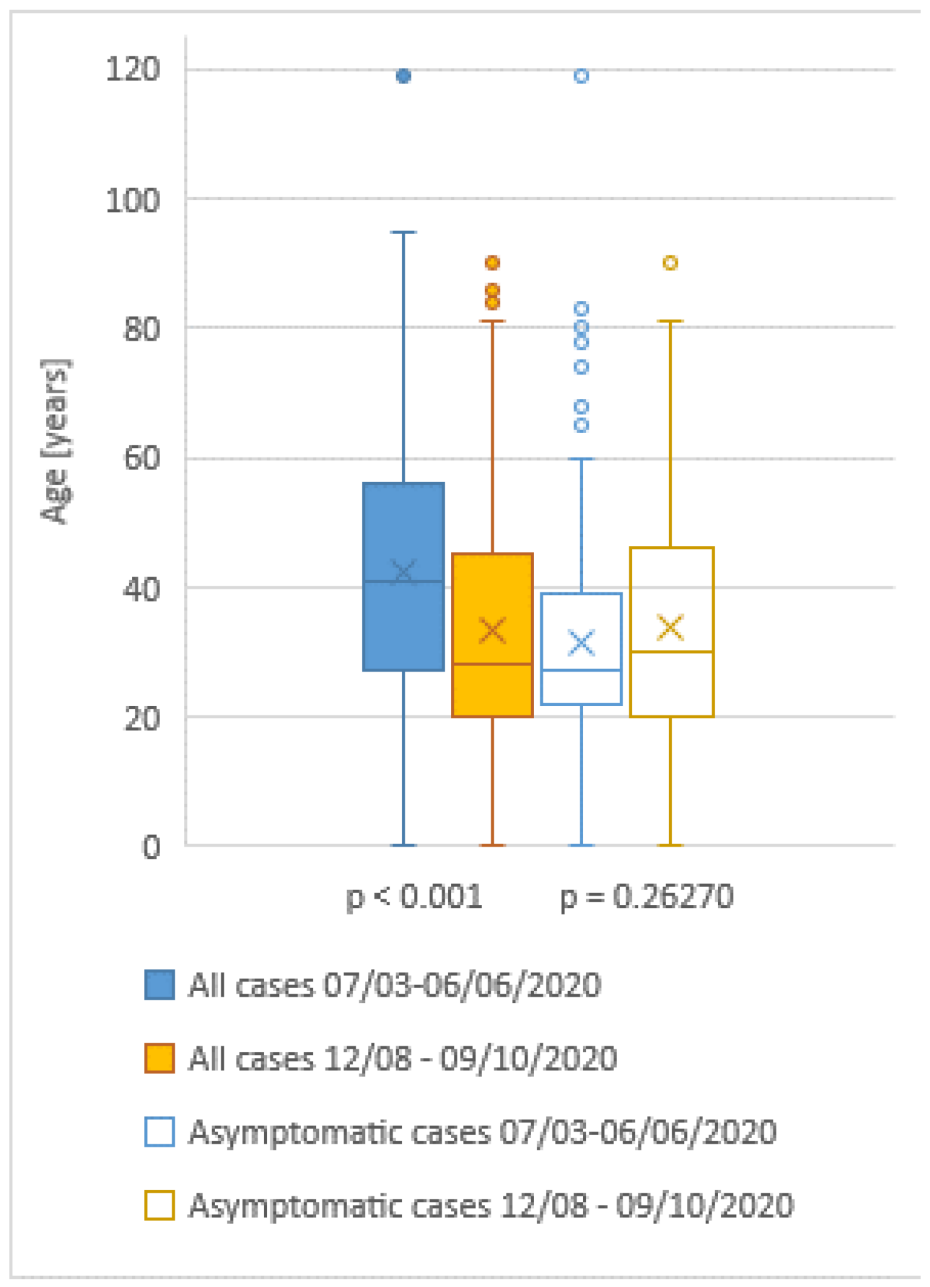
Age range of all cases and asymptomatic cases in the two periods

The proportion of asymptomatic persons not reporting at least one symptom (Figure 2 (Fig. 2)) who tested positive for SARS-COV-2 in the second period was 55% (286 of 520 cases), whereas during the first wave from March to June 2020, this percentage was 14.4% (169 of 1,170 cases). The median age of all cases in the first period was 41 years (0–119), and in autumn it was 28 years (0–90; p<0.001; Figure 3 (Fig. 3)). Comparing the median age of asymptomatic cases, there is no statistically significant difference: median age 27 years (0–119) in spring versus 30 years (0–90) in autumn (p=0.26270).

**Figure 4:**
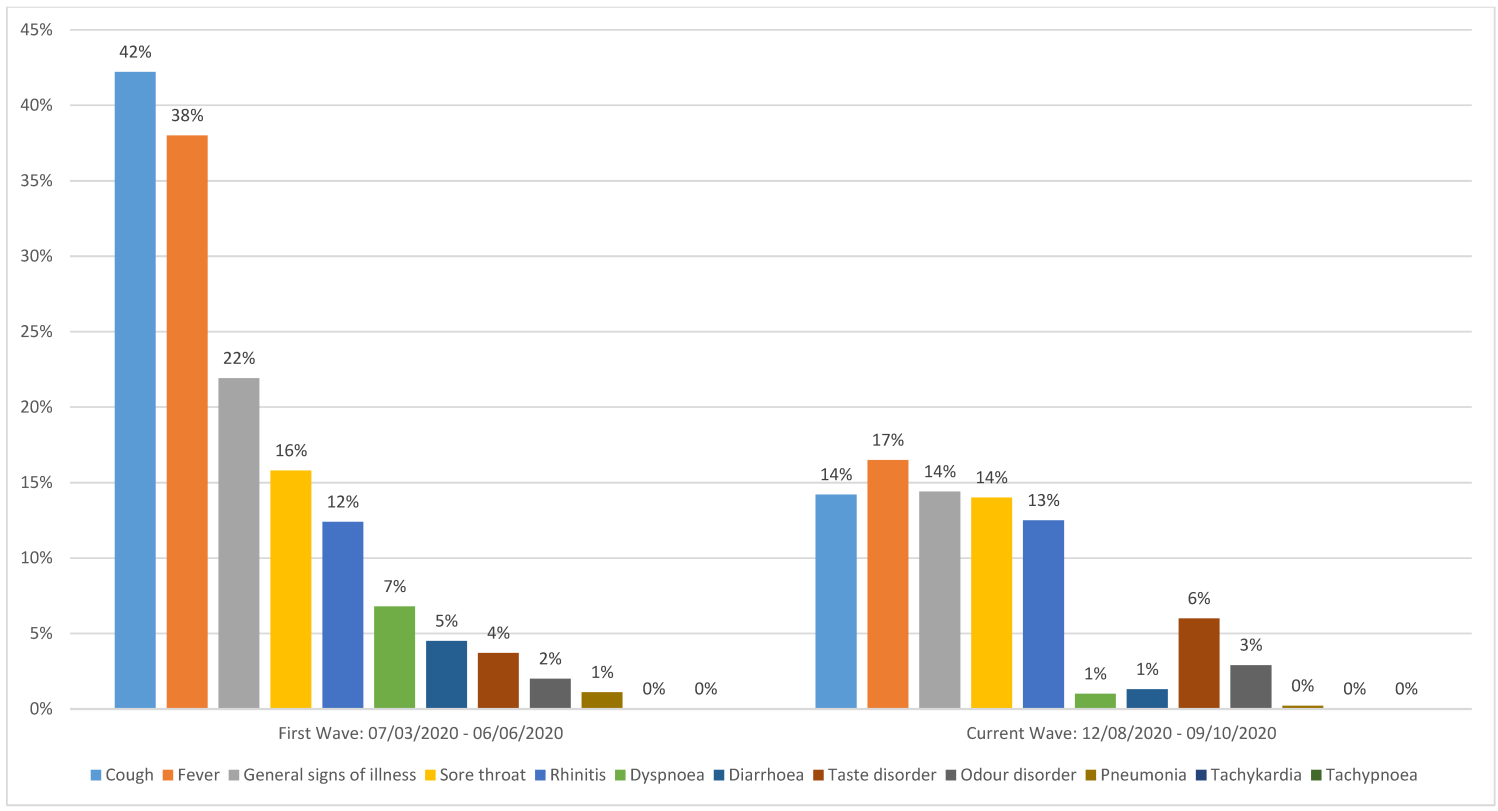
Proportion of reported symptoms during case investigation

A comparison of symptoms (Figure 4 (Fig. 4)) shows that the most common symptoms of COVID-19 in the first phase (cough, fever and general feeling ill) were less often reported in the second wave: cough 14% vs. 42%, fever 17% vs. 38%, general feeling of illness 14% vs. 22%. Pneumonia was reported in only one case out of 520 in the second period, whereas in spring, 13 cases initially exhibited pneumonia (1%). The case fatality rate was 0.2% for the second period compared to 2.1% in spring. 

## Discussion

Our analysis of the two periods demonstrates a change in epidemiological characteristics. The proportion of asymptomatic cases in the second period is large, not only in comparison to the first wave in Regensburg, but also in comparison to other studies [10], [11]. The rate of overall positive PCR results has decreased from around 10% during the first wave to below 1% for the second period in Germany, during which the total number of performed tests has multiplied [12]. In our collective, the median age of all cases is lower in the second period, but the age of asymptomatic cases is not different. It can be concluded that more people of younger age have become infected. Moreover, it can be assumed that a higher percentage of real infections is detected compared to the first wave (less under-reporting). A different age distribution among the infected together with a lower number of unknown infections might be the cause of the lower CFR during the second period to date. 

On the other hand, fever and cough were the leading symptoms in all age groups in Regensburg during the first wave (data from a case series of 1,084 consecutive cases, not yet published). It is not clear why these symptoms are reported in such a low proportion in the second period. 

One possible explanation is that the high number of asymptomatic cases reflects a relevant proportion of uninfected or non-contagious persons, respectively. Accordingly, from August onwards, possible false-positive test results (with high ct values above 35 or only one amplified gene) in asymptomatic tested persons have been recognized more often. This problem might be partially due to testing in a population with a low pre-test probability, high test volumes, and heterogeneous laboratory standards. Thus, the public health authorities are confronted with varying result quality, complicating effective containment. Without clinical information on symptoms, it is more difficult to evaluate the relevance of borderline PCR findings.

Our analysis is limited by the preliminary nature of data, the retrospective design, and a record of symptoms reported by the cases themselves (interview/recall bias). 

In conclusion, the epidemiology in the second period is different from the first wave. We observed a high proportion of asymptomatic cases with a considerable proportion of high ct values in PCR tests. It is difficult to decide whether these results represent infections in the later period that were previously missed or false positive results, and sheds doubt on strategies of high-volume testing in populations with low pre-test probability. Testing should be prioritized for indications with adequate pre-test probability, such as symptomatic cases, close contact persons and outbreak examinations. Pool-testing and targeted testing strategies should be evaluated. Testing strategies with on-demand or free-of-charge tests of asymptomatic persons in very low incidence situations can lead to a low positive predictive value of the tests and place a high burden on finite capacities.

## Notes

### Competing interests

The authors declare that they have no competing interests.
